# The latest trends in nanophotonics

**DOI:** 10.1515/nanoph-2022-0191

**Published:** 2022-04-27

**Authors:** Minkyung Kim, Namkyoo Park, Hak Joo Lee, Junsuk Rho

**Affiliations:** Department of Mechanical Engineering, Pohang University of Science and Technology (POSTECH), Pohang 37673, Republic of Korea; Photonic Systems Laboratory, Department of Electrical and Computer Engineering, Seoul National University, Seoul 08826, Republic of Korea; Center For Advanced Meta-Materials and Division of Applied Nanomechanics, Korea Institute of Machinery and Materials (KIMM), Daejon 34103, Republic of Korea; Department of Chemical Engineering, Pohang University of Science and Technology (POSTECH), Pohang 37673, Republic of Korea; POSCO-POSTECH-RIST Convergence Research Center for Flat Optics and Metaphotonics, Pohang 37673, Republic of Korea

Recent explosive growth in nanophotonics, ignited by the rapidly developing nanotechnologies, has demonstrated that light exhibits extraordinary light–matter interactions with subwavelength-scale structures. Such exotic light behaviors not only manifest the importance of searching for unprecedented optics but also suggest the possibility of real-world applications in the visible range. Indeed, recent progress in nanophotonics has revealed that nanophotonic-based devices and applications can be powerful candidates for replacing the conventional bulky optical components in a compact manner.

*The International Conference on Metamaterials, Photonic Crystals, and Plasmonics* (META) is an annual conference for nanophotonics research. It especially covers a variety of research in metamaterials, photonic crystals, plasmonics, and nanophotonic devices and applications. The latest conference, META’21, was held online due to the COVID-19 pandemic (July 20–23, 2021), where the latest developments in nanophotonics, metamaterials, and related topics were livestreamed across the world. This special issue “The latest trends in nanophotonics” introduces a collection of invited and selected research and review articles from the conference.

Plasmonics is one major branch of nanophotonics, dealing with the surface plasmons, i.e., collective oscillation of electrons at metal–dielectric interfaces. Kim et al. [1] review plasmonics in ultrashort time scale (∼femtoseconds or less), the so-called ultrafast plasmonics. Fundamentals and recent achievements in ultrafast plasmonics are reviewed extensively in two selected examples: strong-field physics and ultraprecision spectroscopy. Menabde et al. [2] present a comprehensive review on image polaritons, a new type of the polaritonic modes coupled with its mirror image when the material is near a highly conductive material, formed in van der Waals crystals. The authors describe the dispersion of the image polaritons and those in a variety of van der Waals crystals including the hyperbolic and nonlocal characteristics and experimental breakthroughs. Xu et al. [3] propose an equilibrium-thermodynamic computational method to generalize the previously reported theories to compute plasmoelectric potential. To improve the application scope and the accuracy of the previous model, the authors introduce an equivalent wavelength method to estimate absorption cross-section and incorporate the plasmonic local heating. The generalized method can quantify the plasmoelectric potential in non-Mie-resonant plasmonic systems whereas the conventional method is only applicable to the Mie-resonant systems.

Two main features of plasmonics are tight field confinement and field enhancement. The former enables a hidden light–matter interaction [4]. Sakai et al. demonstrate that plasmonic nanostructures consisting of gold tetramers can squeeze structured light with a quadrupolar profile within a nanoscale area. This structured light tightly confined in plasmonic nanostructures allowed the authors to access multipolar transitions that were otherwise forbidden due to the length scale mismatch. Meanwhile, the latter, i.e., the field enhancement near plasmonic nanostructures, is known to accelerate the spontaneous emission of nearby emitters but simultaneously exhibit a quenching effect. Baghramyan and Ciracì [5] use quantum hydrodynamic theory to evaluate the fluorescence enhancement of the emitter and contradict the recently reported monotonically increasing fluorescence enhancement in plasmonic nanocavities. By including the nonlocality and electron tunneling, this description predicts the quenching of the fluorescence still exists.

This issue also presents implementations of artificial intelligence and inverse design with nanophotonics. Advantages, challenges, and perspectives of state-of-the-art optimization methods, nanophotonic inverse design, artificial intelligence, and the latest hybrid techniques are reviewed comprehensively by Wang et al. [6]. Applying deep learning in nanophotonics can be particularly promising in realizing device applications. Gao et al. [7] report a review article about deep learning approaches toward computational spectrometers. This new type of spectrometers, in which the spectral information is recovered by nanophotonic-based computational techniques, is introduced in detail, along with the adoption of deep learning approaches in inverse design problems. Bayati et al. [8] demonstrate an extended depth of focus metaoptics exhibiting a lens-like point spread function and a high focusing efficiency by using inverse design techniques. This article also reports full-color imaging across the full visible spectrum enabled by the extended depth of focus metalens, demonstrating the possibilities of nanophotonics in improving imaging quality.

Nanophotonics can be a powerful candidate for improving the functionality and compactness of spectroscopy and image sensor. Recent trends in surface-enhanced infrared absorption spectroscopy including the nanoantenna- and metamaterial-based techniques are intensively reviewed by Tanaka et al. [9]. The nanophotonic-based approaches to overcome the previous problems, i.e., low absorption cross-section and poor signal-to-noise, and advanced materials and methods are introduced. Lee et al. [10] demonstrate the near-infrared spectral imager by integrating the metasurface bandpass filter array into a CMOS image sensor and its hyperspectral imaging as a proof-of-concept. This CMOS-compatible imager is expected to be implemented in practical and portable applications such as mobile devices.

Two review articles are dedicated to intensive studies on some selected nanostructures in photonics. Lee and Kim [11] review nanowires, which can be deployed as a resonator and waveguide in nanophotonics and optoelectronics, and their applications. This review article also discusses the combination of nanowires and two-dimensional materials. The other is the gyroid structures self-assembled by block copolymers, i.e., block copolymer gyroids [12]. Park et al. review the characteristics of the lattice, its optical properties in nanophotonics, and its applications in topological photonics in detail and provide remaining challenges and perspectives for future research.

In addition, this issue contains three review articles on nanophotonics-enabled devices and applications. Lin et al. [13] discuss the optical beam steering in active metasurface, slow light waveguides, and optical phase arrays. Whereas conventional devices are bulky and have speed and reliability issues, these nanophotonic-based devices provide efficient and fast control of light with smaller device sizes and good far-field resolution. Qin et al. [14] present a comprehensive review on magneto-optical materials and their device applications in nanophotonics. Magneto-optical phenomena in magnetoplasmonics and all-dielectric nanostructures are introduced in detail, along with their potential applications for sensing and realizing nonreciprocity. Lu et al. [15] review photonics-driven approaches towards terahertz generation and detection beyond short-carrier-lifetime semiconductors. Diode junctions, plasmonic nanostructures, ultrafast spintronics, and low-dimensional materials for ultrafast carrier dynamics are discussed. These terahertz photoconductors and photomixers are emerging as alternatives to short-carrier-lifetime semiconductors.

Developing advanced fabrication techniques for nanostructures is also an important job. Kim et al. [16] report two manufacturing methods, transfer printing and soft nanoimprinting, to fabricate silver nanostructures by mechanically patterning ionic silver ink coating. The authors exploit one of the techniques to fabricate mechanically tunable polarization-sensitive color filters by patterning silver nanostructures on an elastomer mold.

Metasurfaces, two-dimensional arrays of nanostructures, are also discussed in this issue. All-dielectric metasurfaces for photoluminescence enhancement are reported by Lin et al. [17]. The collective Mie resonances mediate the inter-unit coupling between unit elements and result in coherence resonance and enhanced photoluminescence intensity. Metasurfaces can be integrated with a conventional optical fiber by patterning the nanostructures on the end-facet of fiber [18]. Ghimire et al. fabricate an asymmetric metasurface on the optical fiber and demonstrate the highly polarization- and wavelength-dependent transmission. This polarization-dependent optical filter will find wide applications in optical fiber imaging and sensing applications.

For practical applications, making the nanophotonic systems dynamically tunable is one of the most challenging yet important tasks. Several efforts toward active nanophotonics are also introduced in this issue. Kim et al. [19] report the experimental demonstration of the tunable metasurface for two-dimensional beam steering in the infrared regime. The metasurface unit cell includes the indium tin oxide sandwiched by the gold mirror and top gold antennas functioning as the electrodes and the electric voltages are assigned to each unit cell individually. This electrically tunable beam steering device will be integrated into real-world applications such as light detection and ranging in near future. The dynamic tunability can be also implemented in nonlinear photonic systems. Zhu et al. [20] present a dynamically tunable nonlinear metasurface by exploiting the phase change of germanium antinomy telluride (GST). A three-level switching of the second harmonic generation is demonstrated using the GST-based hybrid metasurface that features a gap surface plasmon resonance. Meanwhile, scatterers that exhibit different optical responses under mechanical stimuli, so-called mechanoresponsive scatterers, are reviewed by Cho et al. [21]. Various types of scatterers in different dimensions, their fabrication methods, mechanisms, and potential applications are also covered. These mechanoresponsive systems have their unique advantages of fast responses, zero energy consumption, and easy fabrication requirements and are expected to find wide applications.

Other subjects of nanophotonics, such as isospectrality [22], nodal lines [23], and spin Hall effect of light [24] are also covered in this issue. Different photonic systems may have identical spectra; this so-called isospectrality in photonics is reviewed by Park et al. [22]. Two subfields, supersymmetric photonics and interdimensional isospectrality, are discussed in detail. The review article also covers hyperuniformity and machine learning for future research. Park et al. [23] present an extensive summary of nodal lines. Theoretical concepts, computation methods of topological invariants, and realization of nodal lines in both photonic and other physics are introduced, with special emphasis on topological physics. Finally, Kim et al. [24] generalize an explicit analytic formula of the spin Hall effect from linearly polarized light to arbitrarily polarized light. The authors suggest a way to enhance the spin Hall effect of light at any incident angle by varying polarization and explain interface independence under circular polarization.

This special issue provides a good overview of research and developments in the dynamic fields of nanophotonics. We are grateful for all the efforts and contributions from the authors and hope that this special issue will serve as inspiration for other scholars.
